# On Gap-Based Lower Bounding Techniques for Best-Arm Identification

**DOI:** 10.3390/e22070788

**Published:** 2020-07-20

**Authors:** Lan V. Truong, Jonathan Scarlett

**Affiliations:** 1 Department of Engineering, University of Cambridge, Cambridge CB2 1PZ, UK; 2 Department of Computer Science & Department of Mathematics, National University of Singapore, Singapore 117418, Singapore; scarlett@comp.nus.edu.sg

**Keywords:** multi-armed bandits, best-arm identification, information-theoretic lower bounds, PAC learning

## Abstract

In this paper, we consider techniques for establishing lower bounds on the number of arm pulls for best-arm identification in the multi-armed bandit problem. While a recent divergence-based approach was shown to provide improvements over an older gap-based approach, we show that the latter can be refined to match the former (up to constant factors) in many cases of interest under Bernoulli rewards, including the case that the rewards are bounded away from zero and one. Together with existing upper bounds, this indicates that the divergence-based and gap-based approaches are both effective for establishing sample complexity lower bounds for best-arm identification.

## 1. Introduction

The multi-armed bandit (MAB) problem [1] provides a versatile framework for sequentially searching for high-reward actions, with applications including clinical trials [2], online advertising [3], adaptive routing [4], and portfolio design [5]. The best-arm identification problem seeks to find the arm with the highest mean using as few arm pulls as possible, and dates back to the works of Bechhofer [6] and Paulson [7]. More recently, several algorithms have been proposed for best-arm identification, including successive elimination [8], lower-upper confidence bound algorithms [9,10], PRISM [11], and gap-based elimination [12]. The latter establishes a sample complexity that is known to be optimal in the two-arm case [13], and more generally near-optimal.

Complementary to these upper bounds is information-theoretic lower bounds on the performance of any algorithm. Such bounds serve as a means to assess the degree of optimality of practical algorithms, and identify where further improvements are possible, thus focusing research towards directions that can have the greatest practical impact. Lower bounds were given by Mannor and Tsitsiklis [14] for Bernoulli bandits, and by Kaufmann et al. [15] for more general reward distributions. Both of these works were based on the difficulty of distinguishing bandit instances that differ in only a single arm distribution, but the subsequent analysis techniques differed significantly, with [14] using a direct change-of-measure analysis and introducing gap-based quantities equaling the difference between two arm means, and [15] using a form of the data processing inequality for KL divergence. We refer to these as the gap-based and divergence-based approaches, respectively. Further works on best-arm identification lower bounds include [16,17,18].

The divergence-based approach was shown in [15] to attain a stronger result than that of [14] with a simpler proof, as we outline in Section 2.2. In this paper, we address the question of whether the gap-based approach is fundamentally limited, or can be refined to attain a similar results to [15]. We show that the correct answer is the latter in many cases of interest, by suitable refinements of the analysis of [14]. The existing results and our results are presented in Section 2, and our analysis is presented in Section 3.

## 2. Overview of Results

### 2.1. Problem Setup

We consider the following setup:

- There are *M* arms with Bernoulli rewards; the means are $p=(p_{1},p_{2},\cdots ,p_{M})$, and this set of means is said to define the bandit instance. Our analysis will consider instances with arms sorted such that $p_{1}\geq p_{2}\cdots \geq p_{M}$, without loss of generality.
- The agent would like to find an arm whose arm mean is within ϵ of the highest arm mean for some 0<ε<1, i.e., $p_{l}>p_{1}-\varepsilon$. Even if there are multiple such arms, just identifying one of them is good enough.
- In each round, the agent can pull any arm l∈[M] and observe an reward $X_{l}^{\left(s\right)}∼Bernoulli\left(p_{l}\right)$, where *s* is the number of times the *l*-th arm has been pulled so far. We assume that the rewards are independent, both across arms and across times.
- In each round, the agent can alternatively choose to terminate and output an arm index $\hat{l}$ believed to be ϵ-optimal. The index at which this occurs is denoted by *T*, and is a random variable because it is allowed to depend on the rewards observed. We are interested in the expected number of arm pulls (also called the sample complexity) $E_{p}\left[T\right]$ for a given instance p, which should ideally be as low as possible.
- An algorithm is said to be (ε,δ)-PAC (Probably Approximately Correct) if, for all bandit instances, it outputs an ε-optimal arm with probability at least 1−δ when it terminates at the stopping time *T*.

We will frequently make use of some fundamental quantities. First, the best arm mean and the gap to the best arm are denoted by

(1) $$\begin{array}{cc} p_{*} & :=p_{1}, \end{array}$$

(2) $$\begin{array}{cc} \Delta_{l} & :=p_{*}-p_{l}. \end{array}$$

The set of ϵ-optimal arms and the set of ϵ-suboptimal arms are respectively given by

(3) $$M(p,\varepsilon ):=\{l\in \left[M\right]:p_{l}>p_{*}-\varepsilon \},$$

(4) $$N(p,\varepsilon ):=\{l\in \left[M\right]:p_{l}\leq p_{*}-\varepsilon \},$$

and we make use of the binary KL divergence function

(5) $$\mathrm{KL}\left(p,q\right):=p\log\frac{p}{q}+\left(1-p\right)\log\frac{1-p}{1-q},$$

where here and subsequently, log(·) denotes the natural logarithm.

### 2.2. Existing Lower Bounds

For any fixed $\underline{p}\in \left(0,1/2\right)$, Mannor and Tsitsiklis [14] showed that if an algorithm is (ϵ,δ)-PAC with respect to all instances with $\min_{l}p_{l}\geq \underline{p}>0$, and if $\epsilon \leq \frac{1-p^{*}}{4}$ and $\delta \leq e^{-8}/8$, then for any constant α∈(0,2), there exists $c_{1}=O\left({\underline{p}}^{2}\right)$ (depending on α) such that

(6) $$\begin{array}{c} E_{p}\left[T\right]\geq c_{1}\left[\frac{{\left(|\tilde{M}\left(p,\varepsilon \right)|-1\right)}^{+}}{\varepsilon^{2}}+\sum_{l\in \tilde{N}\left(p,\varepsilon \right)}\frac{1}{\Delta_{l}^{2}}\right]\log\frac{1}{8\delta } \end{array}$$

where

(7) $$\begin{array}{c} \tilde{M}\left(p,\varepsilon \right)=M\left(p,\varepsilon \right)\cap \left\{l\in \left[M\right]:p_{l}\geq \frac{\varepsilon +p_{*}}{2-\alpha }\right\}, \end{array}$$

(8) $$\begin{array}{c} \tilde{N}\left(p,\varepsilon \right)=N\left(p,\varepsilon \right)\cap \left\{l\in \left[M\right]:p_{l}\geq \frac{\varepsilon +p_{*}}{2-\alpha }\right\}. \end{array}$$

Note that the subsets $\tilde{M}\left(p,\varepsilon \right)$ and $\tilde{N}\left(p,\varepsilon \right)$ do not always form a partition of the arms, i.e., it may hold that $\tilde{M}\left(p,\varepsilon \right)\cup \tilde{N}\left(p,\varepsilon \right)⊊\left[M\right]$. The sets increase in size as α decreases, but implicitly this leads to a lower value of $c_{1}$. In addition, as we will see below, the ${\underline{p}}^{2}$ dependence entering via $c_{1}$ is not necessary.

We also note that the lower bound in (6) depends on the instance-specific quantities $\tilde{M}\left(p,\varepsilon \right)$, $\tilde{N}\left(p,\varepsilon \right)$, and $\Delta_{l}$, and is thus an instance-dependent bound. On the other hand, the lower bound is only stated for (ε,δ)-PAC algorithms, and the PAC guarantee requires the algorithm to eventually succeed on any instance (subject to the assumptions given on $p_{l}$, ϵ, and δ).

Kaufmann et al. [15] improved Mannor and Tsitsiklis’s lower bound by using a form of data processing inequality for KL divergence, leading to the following whenever δ≤0.15 and $0<\varepsilon <\min\{p_{*},1-p_{*}\}$ [15] (Remark 5):

(9) $$\begin{array}{c} E_{p}\left[T\right]\geq \left[\frac{|M(p,\varepsilon )|-1}{\mathrm{KL}(p_{*}-\varepsilon ,p_{*}+\varepsilon )}+\sum_{l\in N(p,\varepsilon )}\frac{1}{\mathrm{KL}(p_{l},p_{*}+\varepsilon )}\right]\log\frac{1}{2.4\delta }. \end{array}$$

To directly compare this result with (6), it is useful to apply the following inequality [19] (Equation (2.8)):

(10) $$\begin{array}{c} 2{\left(p-q\right)}^{2}\leq \mathrm{KL}\left(p,q\right)\leq \frac{{\left(p-q\right)}^{2}}{q(1-q)}, \end{array}$$

which yields

(11) $$\begin{array}{c} E_{p}\left[T\right]\geq \left(p^{*}+\epsilon \right)\left(1-p^{*}-\epsilon \right)\left[\frac{|M(p,\varepsilon )|-1}{4\epsilon^{2}}+\sum_{l\in N(p,\varepsilon )}\frac{1}{{\left(\varepsilon +\Delta_{l}\right)}^{2}}\right]\log\frac{1}{2.4\delta }. \end{array}$$

Even this weakened bound can significantly improve on (6), since (i) $M\left(p,\varepsilon \right)⊃\tilde{M}\left(p,\varepsilon \right)$ and $N\left(p,\varepsilon \right)⊃\tilde{N}\left(p,\varepsilon \right)$, (ii) the ${\underline{p}}^{2}$ dependence is replaced by $\left(p^{*}+\epsilon \right)\left(1-p^{*}-\epsilon \right)$, so the dependence on the smallest arm mean is avoided (The $1-p^{*}-\epsilon$ term is potentially small when ϵ is close to $1-p^{*}$, but since (6) assumes $\epsilon \leq \frac{1-p^{*}}{4}$, we can still say that (11) is at least as good as (6)), and (iii) the assumption $\epsilon \leq \frac{1-p^{*}}{4}$ is avoided.

### 2.3. Our Result and Discussion

Our lower bound, stated in the following theorem, is developed based on Mannor and Tsitsiklis’s analysis for best-arm identification [14] (Theorem 1), but uses novel refinements of the techniques therein to further optimize the bound (see Appendix C for an overview of these refinements).

**Theorem** **1.** For any bandit instance $p\in {\left(0,p_{*}\right]}^{M}$ with $p_{*}\in \left(0,1\right)$, and any (ε,δ)-PAC algorithm with $0<\varepsilon <1-p_{*}$ and $0<\delta <\delta_{0}$ for some $\delta_{0}<1/4$, we have

(12) $$\begin{array}{cc} E_{p}\left[T\right] & \geq \frac{2\gamma_{0}\left(p_{*}+\varepsilon \right)\left(1-p_{*}-\varepsilon \right)}{7(\xi +1)}\left[\frac{|M(p,\varepsilon )|-1}{4\varepsilon^{2}}+\sum_{l\in N(p,\varepsilon )}\frac{1}{{\left(\varepsilon +\Delta_{l}\right)}^{2}}\right]\log\frac{1+4\delta_{0}}{4\delta }, \end{array}$$

*where*

(13) $$\begin{array}{cc} \gamma_{0} & =\frac{1-4\delta_{0}}{8}, \end{array}$$

(14) $$\begin{array}{cc} \theta & =\frac{2\delta }{1-4\gamma_{0}}=\frac{4\delta }{1+4\delta_{0}}, \end{array}$$

*and ξ>0 is the unique positive solution of the following quadratic equation:*

(15) $$\begin{array}{c} 7\gamma_{0}\xi^{2}\log\frac{1}{\theta }=3\left(\xi +1\right). \end{array}$$

Observe that this result matches (11) (with modified constants), and therefore exhibits the above benefit of depending on the full sets M and N without the condition $p_{l}\geq \frac{\varepsilon +p_{*}}{2-\alpha }$ (see (7)–(8)), as well as avoiding the dependence on $\underline{p}$, and permitting the broadest range of ϵ and δ among the above results.

The result (11) in turn matches (9) whenever the right-hand inequality in (10) is tight (i.e., whenever $\mathrm{KL}\left(p,q\right)=\Theta \left(\frac{{\left(p-q\right)}^{2}}{q(1-q)}\right)$). This is clearly true when *p* and *q* (representing the arm means) are bounded away from zero and one, and also in certain limiting cases approaching these endpoints (e.g., when *p* and *q* both tend to one, but $\frac{1-p}{1-q}=\Theta \left(1\right)$). However, there are also limiting cases where the upper bound in (10) is not tight (e.g., $p=1-\sqrt{\eta }$ and q=1−η as η→0), and in such cases, the bound (9) remains tighter than that of Theorem 1.

## 3. Proof of Theorem 1

We follow the general steps of (Theorem 5 [14]), but with several refinements to improve the final bound. The main differences are outlined in Appendix C.

*Step 1: Defining a Hypothesis Test*

Let us denote the true (unknown) expected reward of each arm by $Q_{l}$ for all l∈[M]. Similarly to [14,15], we consider *M* hypotheses as follows:

(16) $$\begin{array}{c} H_{1}:Q_{l}=p_{l}, \end{array}\;\begin{array}{c} \forall l\in \left[M\right], \end{array}$$

and for each l≠1,

(17) $$\begin{array}{c} H_{l}:Q_{l}=p_{*}+\varepsilon , \end{array}\;\begin{array}{c} Q_{l^{\prime }}=p_{l^{\prime }} \end{array}\;\begin{array}{c} \forall l^{\prime }\in \left[M\right]\backslash \left\{l\right\}. \end{array}$$

If hypothesis $H_{l}$ is true, the (ϵ,δ)-PAC algorithm must return arm *l* with probability at least 1−δ. We will bound sample complexity when the hypothesis $H_{l}$ is true. We denote by $E_{l}$ and $P_{l}$ the expectation and probability, respectively, under hypothesis $H_{l}$.

Let $B_{l}$ be the event that the algorithm returns arm *l*. Since $\sum_{l\in M(p,\varepsilon )}P_{1}\left(B_{l}\right)\leq 1$ and |M(p,ε)|≥1, there is at most one arm $l_{0}\in M\left(p,\varepsilon \right)$ that satisfies $P_{1}\left(B_{l_{0}}\right)>\frac{1}{2}$. Defining

(18) $$\begin{array}{c} M_{0}\left(p,\varepsilon \right):=\left\{l\in M\left(p,\varepsilon \right):P_{1}\left[B_{l}\right]\leq \frac{1}{2}\right\}=\left\{l\in M\left(p,\varepsilon \right):l\neq l_{0}\right\}, \end{array}$$

it follows that

(19) $$\begin{array}{c} |M_{0}{\left(p,\varepsilon \right)|\geq (|M\left(p,\varepsilon \right)|-1)}^{+}. \end{array}$$

Define

(20) $$\begin{array}{c} T\left(p,\varepsilon \right):=M_{0}\left(p,\varepsilon \right)\cup N\left(p,\varepsilon \right), \end{array}$$

as well as

(21) $$\begin{array}{c} B_{M(p,\varepsilon )}:=\underset{l\in M(p,\varepsilon )}{⋃}B_{l}, \end{array}$$

which is the event that the policy eventually select an arm in the ε-neighborhood of the best arm in [M]. Since the policy is (ε,δ)-correct with $\delta <\delta_{0}$, we must have

(22) $$\begin{array}{c} P_{1}\left[B_{M(p,\varepsilon )}\right]\geq 1-\delta >1-\delta_{0}, \end{array}$$

and it follows from (18) and (22) that

(23) $$\begin{array}{cc} P_{1}\left[B_{l}\right]\; & \leq \max\left\{\delta_{0},1/2\right\} \end{array}$$

(24) $$\begin{array}{cc} \; & =\frac{1}{2} \end{array}$$

for all l∈T(p,ε).

*Step 2: Bounding the Number of Pulls of Each Arm*

Before proceeding, we make some additional definitions:

(25) $$\begin{array}{cc} \alpha_{l} & :=\frac{\varepsilon +\Delta_{l}}{\left(1-p_{l}\right)p_{l}}, \end{array}$$

(26) $$\begin{array}{cc} \beta_{l} & :=\alpha_{l}\sqrt{\frac{1-p_{l}}{1-(p_{*}+\varepsilon )}}, \end{array}$$

(27) $$\begin{array}{cc} {\tilde{\alpha }}_{l} & :=\alpha_{l}-\frac{4}{3}{\left(\alpha_{l}\left(1-p_{l}\right)\right)}^{2}, \end{array}$$

(28) $$\begin{array}{cc} {\tilde{\beta }}_{l} & :=\beta_{l}-\frac{4}{3}{\left(\alpha_{l}\left(1-p_{l}\right)\right)}^{2}, \end{array}$$

The definitions (27) and (28) will only be used for arms with $\frac{\varepsilon +\Delta_{l}}{p_{l}}\leq \frac{1}{2}$, and for such arms, we will establish in the analysis that ${\tilde{\alpha }}_{l}\geq 0$ and ${\tilde{\beta }}_{l}\geq 0$.

We prove the following lemma, characterizing the probability of a certain event in which (i) the number of pulls of some arm l∈T(p,ε) falls below a suitable threshold (event $A_{l}$ below), (ii) a deviation bound holds regarding the number of observed 1’s from pulling arm *l* (event $C_{l}$ below), and (iii) arm *l* is not returned (event $B_{l}^{c}$).

**Lemma** **1.** *For each l∈[M], let $T_{l}$ be the total number of times that arm l is pulled under the (ε,δ)-correct policy. Let $K_{l}=X_{l}^{\left(1\right)}+X_{l}^{\left(2\right)}+\cdots +X_{l}^{\left(T_{l}\right)}$ be the total number of unit rewards obtained from pulling the arm l up to the $T_{l}$-th time. Let*

(29) $$\begin{array}{c} G_{1,l}:=\frac{7p_{l}^{2}\alpha_{l}^{2}{\left(1-p_{l}\right)}^{2}}{2\left(p_{*}+\varepsilon \right)\sqrt{\left(1-p_{l}\right)\left(1-\left(p_{*}+\varepsilon \right)\right)}}T_{l}, \end{array}$$

(30) $$\begin{array}{c} G_{2,l}:=\left[{\tilde{\beta }}_{l}\left(p_{l}T_{l}-K_{l}\right)1\left\{p_{l}T_{l}>K_{l}\right\}+{\tilde{\alpha }}_{l}\left(p_{l}T_{l}-K_{l}\right)1\left\{p_{l}T_{l}\leq K_{l}\right\}\right]1\left\{\frac{\varepsilon +\Delta_{l}}{p_{l}}\leq \frac{1}{2}\right\}, \end{array}$$

*where $\alpha_{l}$, ${\tilde{\alpha }}_{l}$, and ${\tilde{\beta }}_{l}$ are defined in *(25)*, *(27)*, and *(28)*, respectively. Let*

(31) $$\begin{array}{c} \nu_{l}:=\left(\xi +1\right)\left(\sqrt{\frac{1-p_{l}}{1-p_{*}-\varepsilon }}\right), \end{array}$$

*where ξ is defined in *(15)*. Define the following events:*

(32) $$\begin{array}{c} A_{l}:=\left\{G_{1,l}\leq \frac{1}{\nu_{l}}\log\frac{1}{\theta }\right\}, \end{array}$$

(33) $$\begin{array}{c} C_{l}=\left\{G_{2,l}\leq \frac{\xi }{\nu_{l}}\left(\sqrt{\frac{1-p_{l}}{1-p_{*}-\varepsilon }}\right)\log\frac{1}{\theta }\right\}, \end{array}$$

(34) $$\begin{array}{c} S_{l}:=A_{l}\cap B_{l}^{c}\cap C_{l}. \end{array}$$

*If l∈T(p,ε) (see *(20)*), then under the condition that*

(35) $$\begin{array}{c} E_{1}\left[G_{1,l}\right]<\frac{\gamma_{0}}{\nu_{l}}\log\frac{1}{\theta }, \end{array}$$

*we have*

(36) $$\begin{array}{c} P_{1}\left[S_{l}\right]>\frac{1-4\gamma_{0}}{2}. \end{array}$$

**Proof.**  See Appendix A. □

Intuitively, $A_{l}$ is the event that the total number of times that arm *l* is pulled is small, and $C_{l}$ is the event that $|p_{l}T_{l}-K_{l}|$ is not too large (since pulling an arm $T_{l}$ times should produce roughly $p_{l}T_{l}$ ones). The lemma indicates that if $E[T_{l}]$ is not too large, then $P[A_{l}\cap B_{l}^{c}\cap C_{l}]$ is lower bounded, and this will ultimately lead to a lower bound on $P[B_{l}^{c}]$, the event of primary interest.

In Lemma 2 below, we will use Lemma 1 to deduce a lower bound on $E_{1}\left[G_{1,l}\right]$, which amounts to a lower bound on the average number of arm pulls by the definition of $G_{1,l}$. Before doing so, we introduce a likelihood ratio that will be used in a change-of-measure argument [14].

For any given time t≥1 and l∈[M], let $T_{l}\left(t\right)$ be the total number of times that arm *l* is pulled by time *t*. Define

(37) $$\begin{array}{c} X_{l}^{T_{l}\left(t\right)}:=\left\{X_{l}^{\left(1\right)},X_{l}^{\left(2\right)},\cdots ,X_{l}^{\left(T_{l}\left(t\right)\right)}\right\}, \end{array}$$

and let

(38) $$\begin{array}{c} F_{t}:=\sigma \left(X_{1}^{T_{1}\left(t\right)},X_{2}^{T_{2}\left(t\right)},\cdots ,X_{M}^{T_{M}\left(t\right)}\right) \end{array}$$

be the σ-algebra generated by $X_{1}^{T_{1}\left(t\right)},X_{2}^{T_{2}\left(t\right)},\ldots ,X_{M}^{T_{M}\left(t\right)}$ for all t=1,2,….

Recall that *T* is the stopping time of the algorithm, and that $T_{l}:=T_{l}\left(T\right)$ for all l∈[M]. Moreover, let $W=F_{T}$ be the entire history up to the stopping time *T*. We define the following likelihood ratio:

(39) $$\begin{array}{c} L_{l}\left(w\right)=\frac{P_{l}\left(W=w\right)}{P_{1}\left(W=w\right)} \end{array}$$

for every possible history *w*. Moreover, we let $L_{l}\left(W\right)$ denote the corresponding random variable. Given the history up to time T−1 (i.e., $F_{T-1}$), the arm reward at time *T* has the same probability distribution under $H_{1}$ and $H_{l}$ unless the chosen arm is arm *l*. Therefore, we have

(40) $$\begin{array}{c} L_{l}\left(W\right)=\frac{{\left(p_{*}+\varepsilon \right)}^{K_{l}}{\left(1-p_{*}-\varepsilon \right)}^{T_{l}-K_{l}}}{p_{l}^{K_{l}}{\left(1-p_{l}\right)}^{T_{l}-K_{l}}}, \end{array}$$

where $K_{l}:=X_{l}^{\left(1\right)}+X_{l}^{\left(2\right)}+\cdots +X_{l}^{\left(T_{l}\right)}$ (or the total number of 1’s in the $T_{l}$ pulls of the arm *l*).

The following proposition presents one of our key technical results towards establishing the lower bound. We use the definitions in (1)–(5), along with (25)–(28).

**Proposition** **1.** Fix the bandit instance p, the parameter $0<\varepsilon <1-p_{*}$, and the history W with corresponding values $K_{l}$ and $T_{l}$. Recalling the definitions of $\alpha_{l}$, ${\tilde{\alpha }}_{l}$, and ${\tilde{\beta }}_{l}$ in *(25)*, *(27)*, and *(28)*, respectively, we have

(41) $$\begin{array}{c} L_{l}\left(W\right)\geq \exp\left(-G_{1,l}-G_{2,l}\right), \end{array}$$

*where $G_{1,l}$ and $G_{2,l}$ are defined in *(29)*–*(30)*.*

**Proof.**  See Appendix B. □

Based on Lemma 1 and Proposition 1, we obtain the following extension of [14] (Lemma 6) lower bounding the average of each $G_{1,l}$; this lower bound will later be translated to a lower bound on the number of arm pulls $T_{l}$.

**Lemma** **2.** *For any arm l∈T(p,ε), the following holds:*

(42) $$\begin{array}{c} E_{1}\left[G_{1,l}\right]\geq \frac{\gamma_{0}}{\nu_{l}}\log\frac{1}{\theta }, \end{array}$$

*where θ and $\nu_{l}$ are defined in *(14)* and *(31)*, respectively.*

**Proof.**  We use a proof by contradiction. Assume that

(43) $$\begin{array}{c} E_{1}\left[G_{1,l}\right]<\frac{\gamma_{0}}{\nu_{l}}\log\frac{1}{\theta }, \end{array}$$

then by Lemma 1, Equation (36) holds. Moreover, by Proposition 1, we have

(44) $$\begin{array}{c} L_{l}\left(W\right)\geq \exp\left(-G_{1,l}-G_{2,l}\right), \end{array}$$

and recalling the definition of $S_{l}$ in (34), it follows from (44) that

(45)Ll(W)1Sl≥exp−G1,l−G2,l1Sl(46)≥exp−1νlξ1−pl1−p*−ε+1log1θ1Sl(47)≥exp−1νl(ξ+1)1−pl1−p*−εlog1θ1Sl

where (46) follows from the definitions in (32)–(33), and (47) follows from the fact that $1-p_{l}\geq 1-p_{*}\geq 1-p_{*}-\varepsilon$ for all l∈[M].

By the choice of $\nu_{l}>0$ given in (31), it holds that

(48) $$\begin{array}{c} \left(\xi +1\right)\left(\sqrt{\frac{1-p_{l}}{1-p_{*}-\varepsilon }}\right)\frac{1}{\nu_{l}}=1. \end{array}$$

Hence, from (47) and (48), we have

(49) $$\begin{array}{c} L_{l}\left(W\right)1_{S_{l}}\geq \theta 1_{S_{l}}=\frac{2\delta }{1-4\gamma_{0}}1_{S_{l}}, \end{array}$$

for all l∈T(p,ε), by the definition of θ in (14).

We are now ready to complete the proof:

(50)Pl[Blc]≥Pl[Sl](51)=El[1Sl](52)=E1Ll(W)1Sl(53)≥E12δ1−4γ01Sl(54)=2δ1−4γ0P1[Sl](55)>2δ1−4γ01−4γ02(56)=δ,

where (50) follows from the definition of set $S_{l}$ in (34), (52) follows by a standard change of measure [20], (53) follows from (49), and (55) follows from (36) of Lemma 1 (recall that we assumed (43)).

The inequality (56) shows a contradiction to the fact that under $H_{l}$, the (ε,δ)-correct bandit policy must return the arm *l* with probability at least 1−δ, i.e., $P_{l}\left(B_{l}^{c}\right)\leq \delta$. This concludes the proof. □

From Lemma 2 and the definition of $G_{1,l}$ in (29), it holds that

(57) $$\begin{array}{c} E_{1}\left[\frac{7\alpha_{l}^{2}p_{l}^{2}{\left(1-p_{l}\right)}^{2}}{2\left(p_{*}+\varepsilon \right)\sqrt{\left(1-p_{l}\right)\left(1-\left(p_{*}+\varepsilon \right)\right)}}T_{l}\right]\geq \frac{\gamma_{0}}{\nu_{l}}\log\frac{1}{\theta } \end{array}$$

for all l∈T(p,ε). Hence, and using the definition of $\nu_{l}$ in (31), we have

(58)E1αl2pl2(1−pl)2Tl≥2γ0(p*+ε)(1−pl)(1−p*−ε)7(1+ξ)1−p*−ε1−pllog1θ(59)=2γ0(p*+ε)(1−p*−ε)7(1+ξ)log1θ.

*Step 3: Deducing a Lower Bound on the Sample Complexity*

For any arm l∈T(p,ε), by the definition of $\alpha_{l}$ in (25), we have

(60) $$\begin{array}{c} \alpha_{l}^{2}p_{l}^{2}{\left(1-p_{l}\right)}^{2}={\left(\varepsilon +\Delta_{l}\right)}^{2}. \end{array}$$

Note that $0\leq \Delta_{l}<\varepsilon$ for all $l\in M_{0}\left(p,\varepsilon \right)$, since $M_{0}\subseteq M$, the set of ϵ-optimal arms. Therefore, we can further simplify (60) to

(61) $$\begin{array}{c} \alpha_{l}^{2}p_{l}^{2}{\left(1-p_{l}\right)}^{2}\leq 4\epsilon^{2} \end{array}$$

for $l\in M_{0}\left(p,\varepsilon \right)$.

Substituting (60)–(61) into (59), we obtain

(62)Ep[T]=Ep∑l=1MTl(63)≥Ep∑l∈M0(p,ε)Tl+Ep∑l∈N(p,ε)Tl(64)≥2γ0(p*+ε)(1−p*−ε)7(ξ+1)|M0(p,ε)|4ε2+∑l∈N(p,ε)1(ε+Δl)2log1θ(65)=2γ0(p*+ε)(1−p*−ε)7(ξ+1)|M0(p,ε)|4ε2+∑l∈N(p,ε)1(ε+Δl)2log1+4δ04δ,

where (65) uses the definition of θ in (14). Finally, we obtain (12) from (65) and (19).

## 4. Conclusion

We have presented a refined analysis of best-arm identification following the gap-based approach of [14], but incorporating refinements that circumvent some weaknesses, leading to a bound matching the divergence-based approach [15] in many cases. It would be of interest to determine whether further refinements could allow this approach to match [15] in all cases, or the extent to which the gap-based approach extends beyond Bernoulli rewards and/or beyond the standard best-arm identification problem (e.g., to ranking problems [21]).

## Appendix A. Proof of Lemma 1 (Constant-Probability Event for Small Enough $E_{1}$[$G_{1,l}$])

As in [14], the proof of this lemma is based on Kolmogorov’s maximum inequality [22].

**Lemma** **A1.**
*(Kolmogorov’s Theorem [22]) Let $Y_{1},Y_{2},\cdots ,Y_{n}:\Omega \to R$ be independent random variables defined on a common probability space (Ω,F,P) with expectation $E[Y_{k}]=0$ and variance $\mathrm{Var}[Y_{k}]<\infty$ for k=1,2,⋯,n. Then, for each λ>0,*

(A1) $$\begin{array}{c} P\left[\max_{1\leq k\leq n}\left|S_{k}\right|\geq \lambda \right]\leq \frac{1}{\lambda^{2}}\mathrm{Var}\left[S_{n}\right]=\frac{1}{\lambda^{2}}\sum_{k=1}^{n}E\left[Y_{k}^{2}\right], \end{array}$$

*where $S_{k}=Y_{1}+Y_{2}+\cdots +Y_{k}$.*

We start by simplifying the main assumption of the lemma:

(A2)γ0νllog1θ>E1G1,l(A3)≥1νllog1θP1G1,l>1νllog1θ(A4)=1νllog1θP1[Alc],

where (A3) follows from Markov’s inequality, and (A4) follows from the definition of $A_{l}$ in (32).

It follows from (A4) that

(A5) $$\begin{array}{c} P_{1}\left[A_{l}\right]\geq 1-\gamma_{0}. \end{array}$$

We define

(A6) $$\begin{array}{c} V=\left\{l\in \left[M\right]:\frac{\varepsilon +\Delta_{l}}{p_{l}}\leq \frac{1}{2}\right\} \end{array}$$

and we will find it convenient to treat the cases l∈V and ł∉V separately. For l∉V, from (30) and (33), we have $A_{l}\cap C_{l}=A_{l}$ since θ∈(0,1),ξ>0, and $G_{2,l}=0$, and it immediately follows from (A5) that

(A7) $$\begin{array}{c} P_{1}\left[A_{l}\cap C_{l}\right]\geq 1-\gamma_{0}. \end{array}$$

On the other hand, for l∈V, we can simplify the definition of ${\tilde{\alpha }}_{l}$ in (27) as follows:

(A8)α˜l=αl−43αl(1−pl)2(A9)=αl−43αl(1−pl)αl(1−pl)(A10)=αl−43αl(1−pl)ε+Δlpl(A11)≥αl−23αl(1−pl)(A12)≥αl−23αl(A13)=13αl(A14)>0,

where (A10) follows from (25), and (A11) follows from the definition of the set V in (A6). It follows that

(A15) $$\begin{array}{c} 0<{\tilde{\alpha }}_{l}\leq \alpha_{l}\leq \beta_{l} \end{array}$$

for all l∈V, where the second inequality in (A15) follows from $p_{l}\leq p_{*}\leq p_{*}+\varepsilon$ and the definitions of $\alpha_{l}$ and $\beta_{l}$ in (25) and (26), respectively.

Similarly, for l∈V, we can simplify ${\tilde{\beta }}_{l}$ from (28) as follows:

(A16)β˜l=βl−43αl(1−pl)2(A17)≥αl−43αl(1−pl)2(A18)=α˜l(A19)>0,

where (A17) follows from (A15), (A18) follows from (27), and (A19) again uses (A15). It follows that

(A20) $$\begin{array}{c} 0<{\tilde{\beta }}_{l}\leq \beta_{l} \end{array}$$

for all l∈V.

Now, let

(A21) $$\begin{array}{c} Z_{l}^{\left(j\right)}:=\beta_{l}\left(X_{l}^{\left(j\right)}-p_{l}\right),\;j=1,2,\cdots \end{array}$$

Then, we have

(A22) $$\begin{array}{cc} E_{1}\left[Z_{l}^{\left(j\right)}\right] & =E_{1}\left[\beta_{l}\left(X_{l}^{\left(j\right)}-p_{l}\right)\right]=\beta_{l}E_{1}\left[X_{l}^{\left(j\right)}-p_{l}\right]=0. \end{array}$$

In addition, we note that $Z_{l}^{\left(1\right)},Z_{l}^{\left(2\right)},\cdots ,$ are a i.i.d. sequence by the i.i.d. property of $X_{l}^{\left(1\right)},X_{l}^{\left(2\right)},\cdots$.

For each positive integer $t_{l}$, let $K_{l,t_{l}}:=\sum_{j=1}^{t_{l}}X_{l}^{\left(j\right)}$, and define

(A23) $$\begin{array}{cc} U_{l} & :=\beta_{l}\left(K_{l}-p_{l}T_{l}\right), \end{array}$$

(A24)Vl(tl):=βl(Kl,tl−pltl)(A25)=∑j=1tlβl(Xl(j)−pl)(A26)=∑j=1tlZl(j).

Observe that

(A27)∑j=1tlE1Zl(j)2=∑j=1tlβl2E1[Xl(j)−pl)2(A28)=∑j=1tlβl2pl(1−pl)(A29)=tlβl2pl(1−pl),

where (A28) follows since a Bernoulli(ρ) variable has variance ρ(1−ρ).

We are now ready to upper bound $P_{1}\left[C_{l}^{c}\cap A_{l}\right]$ for l∈V:

P1[Clc∩Al](A30)=P1β˜l(plTl−Kl)1{plTl>Kl}+α˜l(plTl−Kl)1{plTl≤Kl}>ξνl1−pl1−p*−εlog1θ∩Al(A31)≤P1β˜l|plTl−Kl|1{plTl>Kl}+α˜l|plTl−Kl|1{plTl≤Kl}>ξνl1−pl1−p*−εlog1θ∩Al(A32)≤P1βl|plTl−Kl|>ξνl1−pl1−p*−εlog1θ∩Al(A33)=P1|Ul|>ξνl1−pl1−p*−εlog1θ∩Tl<2(p*+ε)(1−pl)(1−(p*+ε))7νlαl2pl2(1−pl)2log1θ(A34)≤P1maxtl≤2(p*+ε)(1−pl)(1−(p*+ε))log1θ7νlαl2pl2(1−pl)2|Vl(tl)|>ξνl1−pl1−p*−εlog1θ(A35)=P1maxtl≤2(p*+ε)(1−pl)(1−(p*+ε))log1θ7νlαl2pl2(1−pl)2|∑j=1tlZj|>ξνl1−pl1−p*−εlog1θ,

where:

- (A30) uses the definitions of $C_{l}$ and $G_{2,l}$;
- (A32) follows from (A15) and (A20), along with $1\left\{p_{l}T_{l}>K_{l}\right\}+1\left\{p_{l}T_{l}\leq K_{l}\right\}=1$;
- (A33) uses the definitions of $U_{l}$ and $A_{l}$;
- (A34) follows from the definitions of $U_{l}$ and $V_{l}\left(t_{l}\right)$ in (A23) and (A24) (which imply $U_{l}=V_{l}\left(T_{l}\right)$);
- (A35) follows from (A26);

Defining $n_{l}=\frac{2\left(p_{*}+\varepsilon \right)\sqrt{\left(1-p_{l}\right)\left(1-\left(p_{*}+\varepsilon \right)\right)}\log\frac{1}{\theta }}{7\nu_{l}\alpha_{l}^{2}p_{l}^{2}{\left(1-p_{l}\right)}^{2}}$ for brevity, we continue from (A35) as follows:

(A36)P1[Clc∩Al]≤maxtl≤nl∑j=1tlE1Zj2ξνl1−pl1−p*−εlog1θ2(A37)=maxtl≤nlpl(1−pl)tlξνl1−pl1−p*−εlog1θ2βl2(A38)≤2νl(p*+ε)(1−pl)(1−(p*+ε))7ξ21−pl1−p*−εpl(1−pl)log1θβlαl2(A39)=2νl(p*+ε)1−(p*+ε)1−pl7ξ21−pl1−p*−εpllog1θβlαl2(A40)≤3νl1−(p*+ε)1−pl7ξ21−pl1−p*−εlog1θβlαl2(A41)=3νl1−(p*+ε)1−pl7ξ21−pl1−p*−εlog1θ1−pl1−p*−ε(A42)=3(ξ+1)7ξ2log1θ(A43)=γ0,

where:

- (A36) follows from Lemma A1 with $n=n_{l}$ and $k=t_{l}$;
- (A37) follows from (A29);
- (A38) follows from the definition of $n_{l}$;
- (A40) follows since the condition $\frac{\varepsilon +\Delta_{l}}{p_{l}}\leq \frac{1}{2}$ in V yields $\frac{\varepsilon +p_{*}-p_{l}}{p_{l}}\leq \frac{1}{2}$, which implies
  (A44) $$\begin{array}{c} p_{l}\geq \frac{2}{3}\left(p_{*}+\varepsilon \right). \end{array}$$
  for all l∈V;
- (A41) follows from the definitions of $\alpha_{l}$ and $\beta_{l}$ in (25)–(26);
- (A42) follows from the definition of $\nu_{l}$ in (31);
- (A43) follows from the definition of ξ in (15).

Combining (A5) and (A43), it follows that

(A45)P1[Cl∩Al]=P1[Al]−P1[Clc∩Al](A46)≥1−2γ0

for all l∈V, and from (A7) and (A46), we obtain

(A47) $$\begin{array}{c} P_{1}\left[C_{l}\cap A_{l}\right]\geq 1-2\gamma_{0} \end{array}$$

for all l∈T(p,ε).

Finally, recall the definition of $S_{l}$ in (34). From (24) and (A47), and using the union bound, we have

(A48)P1[Sl]>1−2γ0+12(A49)=1−4γ02

for all l∈T(p,ε), as desired.

## Appendix B. Proof of Proposition 1 (Bounding a Likelihood Ratio)

We first state the following lemma, which can easily be verified graphically, or proved using basic calculus.

**Lemma** **A2.**
*For any x∈[0,1), the following holds:*

(A50) $$\begin{array}{c} 1-x\geq \exp\left(-\frac{x}{\sqrt{1-x}}\right). \end{array}$$

To prove Proposition 1, we consider two cases:

- **Case** **1:**$\frac{\varepsilon +\Delta_{l}}{p_{l}}>\frac{1}{2}$. In this case, recalling that $\Delta_{l}=p_{*}-p_{l}$, we have
  (A51) $$\begin{array}{cc} \frac{\varepsilon +p_{*}}{p_{l}} & =\frac{\varepsilon +\Delta_{l}}{p_{l}}+1>\frac{3}{2}>1. \end{array}$$
  On the other hand, since $\varepsilon +p_{l}\leq \varepsilon +p_{*}<1$, we have
  (A52)0<ε+Δl1−pl=ε+p*−pl1−pl(A53)=1−1−(p*+ε)1−pl,
  and applying Lemma A2 gives
  (A54)1−ε−p*1−pl=1−ε+Δl1−pl(A55)≥exp−1−pl1−(p*+ε)ε+Δl1−pl(A56)=exp−ε+Δl(1−pl)(1−(p*+ε)).
  Moreover, by the definition of $\alpha_{l}$ in (25), we have
  (A57) $$\begin{array}{c} \alpha_{l}=\frac{\varepsilon +\Delta_{l}}{\left(1-p_{l}\right)p_{l}}>\frac{1}{2(1-p_{l})}, \end{array}$$
  since $\frac{\varepsilon +\Delta_{l}}{p_{l}}>\frac{1}{2}$. It follows from (A57) that
  (A58) $$\begin{array}{c} \alpha_{l}<2\alpha_{l}^{2}\left(1-p_{l}\right). \end{array}$$
  In addition, again using $\frac{\varepsilon +\Delta_{l}}{p_{l}}>\frac{1}{2}$, we have
  (A59) $$\begin{array}{c} p_{l}<2\left(\varepsilon +\Delta_{l}\right), \end{array}$$
  and hence
  (A60)p*+ε=(ε+Δl)+pl(A61)<3(ε+Δl).
  We can now lower bound the likelihood ratio $L_{l}\left(W\right)$ as follows:
  (A62)Ll(W)=ε+p*plKl1−ε−p*1−plTl−Kl(A63)≥exp−ε+Δl(1−pl)(1−(p*+ε))(Tl−Kl)(A64)≥exp−ε+Δl(1−pl)(1−(p*+ε))Tl(A65)=exp−(p*+ε)(ε+Δl)(p*+ε)(1−pl)(1−(p*+ε))Tl(A66)≥exp−3(ε+Δl)2(p*+ε)(1−pl)(1−(p*+ε))Tl(A67)=exp−3αl2pl2(1−pl)2(p*+ε)(1−pl)(1−(p*+ε))Tl,
  where (A63) follows from (A51) and (A56), (A66) follows from (A61), and (A67) follows by the definition of $\alpha_{l}$ in (25). Hence, (41) holds for this case in which $G_{2,l}=0$ (and also using $3\leq \frac{7}{2}$).
- **Case** **2:**$0\leq \frac{\varepsilon +\Delta_{l}}{p_{l}}\leq \frac{1}{2}$. For this case, we have
  (A68)Ll(W)=ε+p*plKl1−ε−p*1−plTl−Kl(A69)=1+ε+ΔlplKl1−ε+Δl1−plTl−Kl(A70)=1−ε+Δlpl2Kl1−ε+Δlpl−Kl1−ε+Δl1−plTl−Kl(A71)=1−ε+Δlpl2Kl1−ε+Δlpl−Kl1−ε+Δl1−plKl(1−pl)/pl1−ε+Δl1−pl(plTl−Kl)/pl,
  where (A69) follows from $\Delta_{l}=p_{*}-p_{l}$ along with (A53), and (A70) follows since $1-a^{2}=\left(1-a\right)\left(1+a\right)$.
  From (A53), we have
  (A72) $$\begin{array}{c} 0<{\left(\frac{\varepsilon +\Delta_{l}}{1-p_{l}}\right)}^{2}={\left(1-\frac{1-(p_{*}+\varepsilon )}{1-p_{l}}\right)}^{2}\leq 1-\frac{1-(p_{*}+\varepsilon )}{1-p_{l}}<1. \end{array}$$
  Hence, by Lemma A2, we have
  (A73)1−ε+Δl1−pl2≥exp−11−ε+Δl1−pl2ε+Δl1−pl2(A74)≥exp−1−pl1−p*−εε+Δl1−pl2(A75)=exp−1−pl(1−pl)(1−p*−ε)ε+Δl1−pl2,
  where (A74) follows from (A72).
  For the third term in (A71), we proceed as follows:
  1−ε+Δl1−plKl(1−pl)/pl(A76)=1−ε+Δl1−pl2Kl(1−pl)/pl1+ε+Δl1−pl−Kl(1−pl)/pl(A77)≥exp−1−pl(1−pl)(1−(p*+ε))ε+Δl1−pl2Kl(1−pl)pl1+ε+Δl1−pl−Kl(1−pl)/pl(A78)=exp−(1−pl)2pl(1−pl)(1−(p*+ε))ε+Δl1−pl2Kl1+ε+Δl1−pl−Kl(1−pl)/pl(A79)≥exp−3(1−pl)22(p*+ε)(1−pl)(1−(p*+ε))ε+Δl1−pl2Kl1+ε+Δl1−pl−Kl(1−pl)/pl(A80)=exp−3αl2pl2(1−pl)22(p*+ε)(1−pl)(1−(p*+ε))Kl1+ε+Δl1−pl−Kl(1−pl)/pl(A81)≥exp−3αl2pl2(1−pl)22(p*+ε)(1−pl)(1−(p*+ε))Tl1+ε+Δl1−pl−Kl(1−pl)/pl(A82)≥exp−3αl2pl2(1−pl)22(p*+ε)(1−pl)(1−(p*+ε))Tlexp−ε+Δl1−plKl(1−pl)pl(A83)=exp−3αl2pl2(1−pl)22(p*+ε)(1−pl)(1−(p*+ε))Tlexp−ε+ΔlplKl,
  where (A76) uses $1-a^{2}=\left(1-a\right)\left(1+a\right)$, (A77) follows from (A75), (A79) follows from (A44), (A80) follows by definition of $\alpha_{l}$ in (25), (A81) follows from the fact that $K_{l}\leq T_{l}$, and (A82) follows from the fact that ${\left(1+x\right)}^{-y}\geq \exp\left(-xy\right)$ for all 0≤x and y≥0.
  On the other hand, observe that
  (A84) $$\begin{array}{c} {\left(1-\frac{\varepsilon +\Delta_{l}}{p_{l}}\right)}^{-K_{l}}\geq \exp\left[\left(\frac{\varepsilon +\Delta_{l}}{p_{l}}\right)K_{l}\right] \end{array}$$
  since ${\left(1-x\right)}^{-y}\geq \exp\left(xy\right)$ for all 0≤x≤1 and y≥0. It follows from (A83) and (A84) that
  (A85) $$\begin{array}{c} {\left(1-\frac{\varepsilon +\Delta_{l}}{p_{l}}\right)}^{-K_{l}}{\left(1-\frac{\varepsilon +\Delta_{l}}{1-p_{l}}\right)}^{K_{l}\left(1-p_{l}\right)/p_{l}}\geq \exp\left[-\frac{3\alpha_{l}^{2}p_{l}^{2}{\left(1-p_{l}\right)}^{2}}{2\left(p_{*}+\varepsilon \right)\sqrt{\left(1-p_{l}\right)\left(1-\left(p_{*}+\varepsilon \right)\right)}}T_{l}\right], \end{array}$$
  and it follows from (A71) and (A85) that
  (A86) $$\begin{array}{cc} L_{l}\left(W\right) & \geq {\left(1-{\left(\frac{\varepsilon +\Delta_{l}}{p_{l}}\right)}^{2}\right)}^{K_{l}}{\left(1-\frac{\varepsilon +\Delta_{l}}{1-p_{l}}\right)}^{\left(p_{l}T_{l}-K_{l}\right)/p_{l}}\\ & \;\times \exp\left[-\frac{3}{2\left(p_{*}+\varepsilon \right)\sqrt{\left(1-p_{l}\right)\left(1-\left(p_{*}+\varepsilon \right)\right)}}\alpha_{l}^{2}p_{l}^{2}{\left(1-p_{l}\right)}^{2}T_{l}\right]. \end{array}$$
  Now, since $0<{\left(\frac{\varepsilon +\Delta_{l}}{p_{l}}\right)}^{2}\leq \frac{1}{4}=1-\frac{3}{4}$ (since we are in the case $0\leq \frac{\varepsilon +\Delta_{l}}{p_{l}}\leq \frac{1}{2}$), by Lemma A2, we have
  (A87)1−ε+Δlpl2Kl≥exp−43ε+Δlpl2Kl(A88)≥exp−43ε+Δlpl2Kl(A89)=exp−43αl(1−pl)2Kl(A90)=exp43αl(1−pl)2(plTl−Kl)exp−43αl(1−pl)2plTl,
  where (A89) follows from the definition of $\alpha_{l}$ in (25).
  We now consider two further sub-cases:
  (i) If $p_{l}T_{l}>K_{l}$, then we have
    (A91)1−ε+Δl1−pl(plTl−Kl)/pl≥exp−1−pl1−(p*+ε)ε+Δl1−plplTl−Klpl(A92)=exp−1−pl1−(p*+ε)αlplTl−Kl(A93)=exp−βlplTl−Kl,
    where (A91) follows from Lemma A2 along with (A53), and (A93) follows from the definition of $\beta_{l}$ in (26).
  (ii) If $p_{l}T_{l}\leq K_{l}$, then we have
    (A94)1−ε+Δl1−pl(plTl−Kl)/pl≥exp−ε+Δl1−plplTl−Klpl(A95)=exp−αl(plTl−Kl),
    where (A94) follows from the fact that ${\left(1-x\right)}^{y}\geq \exp\left(-xy\right)$ if 1≥x≥0 and y≤0.
  From (A93) and (A95), we obtain
  (A96) $$\begin{array}{c} {\left(1-\frac{\varepsilon +\Delta_{l}}{1-p_{l}}\right)}^{\left(p_{l}T_{l}-K_{l}\right)/p_{l}}\geq \exp\left[-\left(\beta_{l}\left(p_{l}T_{l}-K_{l}\right)1\left\{p_{l}T_{l}>K_{l}\right\}+\alpha_{l}\left(p_{l}T_{l}-K_{l}\right)1\left\{p_{l}T_{l}\leq K_{l}\right\}\right)\right]. \end{array}$$
  Now, from (A86), (A90), and (A96), we have
  Ll(W)≥exp43αl(1−pl)2(plTl−Kl)exp−43αl2pl(1−pl)2Tl×exp−3αl2pl2(1−pl)22(p*+ε)(1−pl)(1−(p*+ε))Tl(A97)×exp−βl(plTl−Kl)1{plTl>Kl}+αl(plTl−Kl)1{plTl≤Kl}=exp−43αl2pl(1−pl)2Tlexp−3αl2pl2(1−pl)22(p*+ε)(1−pl)(1−(p*+ε))Tl(A98)×exp−β˜l(plTl−Kl)1{plTl>Kl}+α˜l(plTl−Kl)1{plTl≤Kl}≥exp−2(p*+ε)αl2pl2(1−pl)2Tlexp−3αl2pl2(1−pl)22(p*+ε)(1−pl)(1−(p*+ε))Tl(A99)×exp−β˜l(plTl−Kl)1{plTl>Kl}+α˜l(plTl−Kl)1{plTl≤Kl}≥exp−7αl2pl2(1−pl)22(p*+ε)(1−pl)(1−(p*+ε))Tl(A100)×exp−β˜l(plTl−Kl)1{plTl>Kl}+α˜l(plTl−Kl)1{plTl≤Kl},
  where (A98) follows from the definitions of ${\tilde{\alpha }}_{l}$ and ${\tilde{\beta }}_{l}$ in (27)–(28), (A99) follows by writing $\frac{4}{3}\alpha_{l}^{2}p_{l}\left(1-p_{l}^{2}\right)=\frac{4}{3p_{l}}\alpha_{l}^{2}p_{l}^{2}\left(1-p_{l}^{2}\right)$ and applying (A44), and (A100) uses $\sqrt{\left(1-p_{l}\right)\left(1-p^{*}+\epsilon \right)}\leq 1$. Hence, (41) also holds in this case, and the proof is complete.

## Appendix C. Differences in Analysis Techniques

Here we briefly overview some of the main differences in our analysis techniques compared to [14], leading to the improvements highlighted in Section 2:

- We remove the restriction $p_{l}\geq \frac{\varepsilon +p_{*}}{1+\sqrt{\frac{1}{2}}}$ (or $\frac{\varepsilon +\Delta_{l}}{p_{l}}\leq \frac{1}{\sqrt{2}}$) used in the subsets M(p,ε) and N(p,ε) in (Equations (4) and (5) [14]), so that our lower bound depends on all of the arms. To achieve this, our analysis frequently needs to handle the cases $\frac{\varepsilon +\Delta_{l}}{p_{l}}>\frac{1}{2}$ and $\frac{\varepsilon +\Delta_{l}}{p_{l}}\leq \frac{1}{2}$ separately (e.g., see the proof of Proposition 1).
- The preceding separation into two cases also introduces further difficulties. For example, our definition of $G_{2,l}$ in (30) is modified to contain different constants for the cases $p_{l}T_{l}>K_{l}$ and $p_{l}T_{l}\leq K_{l}$, which is not the case in (Lemma 2 [14]). Accordingly, the quantities ${\tilde{\alpha }}_{l}$ in (27) and ${\tilde{\beta }}_{l}$ in (28) appear in our proof but not in [14].
- We replace the inequality ${\left(1-x\right)}^{y}\geq e^{-1.78xy}$ (for $x\in \left(0,\frac{1}{\sqrt{2}}\right)$ and y≥0) (Lemma 3 [14]) by Lemma A2. By using this stronger inequality, we can improve the constant term $c_{1}$ from $O({\underline{p}}^{2})$ to ${\left(p^{*}+\varepsilon \right)}^{2}$. In addition, Lemma A2 does not require the assumption $x\leq \frac{1}{\sqrt{2}}$ as in (Lemma 3 [14]), so we can use it for the case $p_{*}>\frac{1}{2}$, which required a separate analysis in [14].
- To further reduce the constant term from ${\left(p^{*}+\varepsilon \right)}^{2}$ to $\left(p^{*}+\varepsilon \right)$ (see Theorem 1), we also need to use other mathematical tricks to sharpen certain inequalities, such as (A83).
